# Households’ Practices towards Rabies Prevention and Control in Rural Nepal

**DOI:** 10.3390/ijerph20075427

**Published:** 2023-04-06

**Authors:** Alok Dhakal, Ramjee P. Ghimire, Sujit Regmi, Krishna Kaphle

**Affiliations:** 1 Paklihawa Campus, Institute of Agriculture and Animal Science, Tribhuvan University, Bhairahawa 32900, Rupandehi, Nepal; 2 College of Agriculture and Natural Resources, Michigan State University, East Lansing, MI 48824, USA

**Keywords:** zoonosis, prevention, traditional practices, public health, post-exposure prophylaxis

## Abstract

Rabies is a vaccine-preventable, zoonotic, viral disease and a major public health concern for developing countries such as Nepal. A study was conducted from October–December 2021 among 308 household heads from three districts in Nepal (Siraha, Parsa, and Nawalparasi West) through an in-person interview to examine the rural people’s practices towards rabies. Of 70 respondents owning pet animals, 82.9% vaccinated them against rabies but 87.9% (51/58) of them kept a vaccination record. Nearly all respondents (99.7%, 307/308) said they would visit hospitals after being bitten by rabid or rabies suspected animals, and 18.2% (56/308) of them said they would also opt to visit traditional healers seeking treatment against rabies. Seven in ten respondents knew that they should wash bitten body area with soap and water. Around 60% (184/308) of respondents said they would not bother to notify or report to the local authorities if they saw someone bitten by a presumed rabid dog or observed animal behavior suggestive of rabies. The Chi-square test showed a significant association between the socio-demographic characteristics of respondents with practices (good practice and poor practice) towards rabies. The study findings suggest that rural people in Nepal need to be educated with applied rabies control and prevention practices and made aware of health seeking behavior and the role that a community members have to play to control, prevent, and eradicate rabies.

## 1. Introduction

Rabies is a disease of public health and economic importance. Dog-mediated rabies cases, although being vaccine preventable, are still prevalent in all continents except Antarctica [1], and over 90% of human rabies exposures and 99% of deaths occur as a result of contact with rabid dogs. The virus persists mainly in wildlife in developed countries where canine rabies has been eradicated. Rabies virus affects all warm blooded animals and it is fatal once clinical signs are seen [2]. Children under the age of 15 make up 40% of those bitten by suspected rabid animals [1]. The World Health Organization (WHO), along with the Food and Agriculture Organization (FAO), the World Organization for Animal Health (WOAH), and the Global Alliance for Rabies Control (GARC), have launched a collaborative global effort called “United Against Rabies (UAR)” to accomplish the goal of “zero human deaths from dog-mediated rabies by 2030” [3]. It cannot be denied that regular animal vaccination and controlling of stray dogs can decrease cases of human rabies.

Rabies vaccines have been available for over a century, and the majority of deaths occur in countries with inadequate public health services and inaccessible preventive treatment. The FAO [4] reports that rabies is a disease of deprivation and negligence, the mortality from which occurs mostly in resource poor Asian and African countries. When post-exposure prophylaxis (PEP) is given on time along with wound washing, immunization, and Rabies Immune Globulin (RIG), we can be successful in preventing the disease and save the human life [5]. The true number of rabies cases and rabies-related fatalities in humans and animals are underreported [6].

In Nepal, the National Zoonosis Control Program under the Department of Health Services (DHS) has set a goal that no people should die due to unavailability of the anti-rabies vaccine (ARV). It is estimated that nearly half of Nepal’s population is at high risk of contracting rabies, and a quarter is at moderate risk [7]. The number of reported rabies deaths has been fluctuating. According to DHS, in Nepal [7], 18 people died of rabies in 2018/19, 32 persons in 2017/18, eight in 2016/17, six in 2015/16, 13 in 2014/15, and ten in 2013/14.

Several institutions are involved and several activities have been initiated for rabies prevention and control in Nepal [8]; however, not much is known about their effectiveness. In the human health field, the Sukraraj Tropical and Infectious Disease Hospital under the Ministry of Health has a specialized unit for the diagnosis and treatment of rabies or rabies suspected cases. In addition, the Department of Health Services has programs throughout the country to educate people against rabies and provide post exposure care to the people in need. Rabies Immune Globulin (RIG) is available in all seven provincial hospitals free of cost in Nepal. In addition, cell culture vaccines are being used in the entire country [9].

In the animal health sector, the Department of Livestock Services (DLS) provides education to inform pet owners and other domesticated animal owners to manage rabies or rabies suspected cases. The DLS also has a Rabies Vaccine Production Laboratory, which produces the anti-rabies vaccine using cell culture technology [10]. Nationally, the vaccination of pets is done through the government run veterinary hospitals or service centers [11]. However, there are some privately run veterinary clinics that also provide vaccination services. In both cases, pet owners have to pay to vaccinate their pets. Occasionally, some government units, such as municipalities, do campaign for rabies control and prevention programs and provide PEP or pre-exposure prophylaxis (PrEP) vaccination for free.

Human deaths in Nepal due to rabies have been reported in recent years, with possible under-reporting in rural areas of Nepal. The true figure could be many times greater. Scientists have demonstrated that rabies can be successfully controlled and human deaths can be avoided once 70 percent of dogs have been vaccinated [12]. Every year, millions of healthy dogs are killed inhumanely because of the risk of catching rabies [13]. We can eradicate rabies via collaborative efforts, particularly in areas of public awareness and education, which are equally as vital as vaccines, by teaching community people proper practices towards rabies.

In this study, we have examined the practices of people towards rabies, which can be a good source for concerned authorities to design future rabies control and prevention programs and policies.

## 2. Materials and Methods

### 2.1. Study Design

**Figure 1:**
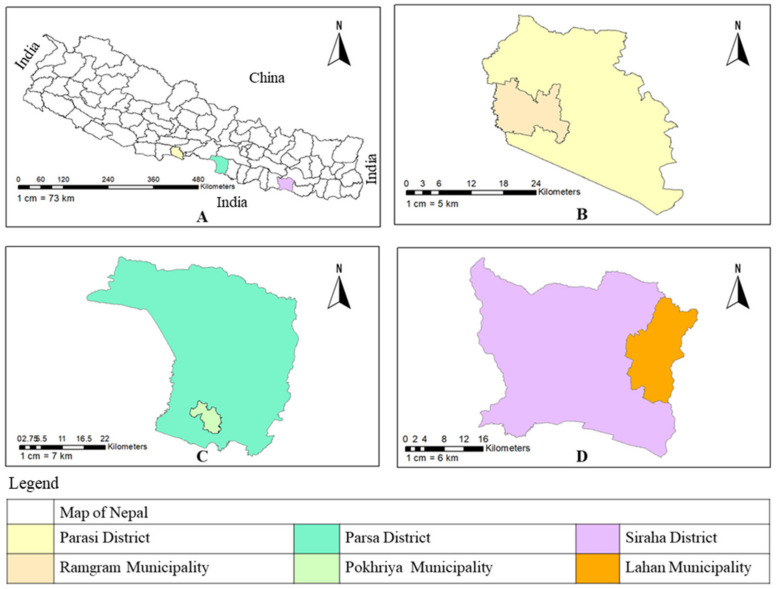
(**A**) Map of Nepal showing study districts. (**B**) Parasi district showing Ramgram municipality. (**C**) Parsa district showing Pokhriya municipality. (**D**) Siraha district showing Lahan municipality.

A cross-sectional study was carried out from October to December 2021 in Siraha, Parsa, and Nawalparasi West (Bardaghat Susta West, called hereafter as Parasi) (Figure 1). From these districts, the municipalities and wards where maximum dog bite cases were observed were selected. The snowball sampling method was employed in the selected three municipalities to identify households and collect data from household head (HH), as done by Tiwari et al. [14] and Tenzin et al. [15]. The survey was carried out among 308 (Siraha: 102, Parsa: 102, Parasi: 104) respondents. In Nepal, decision taking is generally done by household heads; thus, the respondents were HH as in the study viz. Wolelaw et al. [16]. If a HH was absent, the next elderly member in the house was interviewed. Following the selection of the first household, the interviewed HH provided information about the next household until the desired number of respondents had been interviewed.

### 2.2. Procedures and Data Collection Method

In-person interviews were carried out using questionnaires designed by investigators (Supplementary File S1) based on a literature review, researchers’ own experience and knowledge of the subject, and expert consultation. The questionnaires were pre-tested with 15 individuals who were exclusive of the final respondents’ pool. Necessary changes were made in the tools after pre-testing and then the final version of survey was expert validated.

During the interview, the respondents were first explained the purpose and objectives of the study and their oral consent for participation in this study was obtained. Only those who consented to voluntary participation in the study were interviewed. The interview was conducted in Nepali and Hindi languages for the ease of respondents. The research team then recorded the data in English for the hard copy survey.

### 2.3. Measurement of Practices Score

There was a total of eight close-ended practice related questions associated with rabies in the survey. Out of eight, five questions were administered to respondents not owning any pets. For respondents owning pets, an additional three questions were asked. A positive value of one (1) was assigned to each correct answer or a good practice, which was in conformity with existing literatures, and a value of zero (0) was assigned to each wrong answer or a bad practice. Then, the average score of respondents was calculated. For pet owners it was 6.7, and for non-pet owners it was 3.4. Based on the scale’s average score, a binary result was generated. Respondents who received above-average scores were thought to have good practice, while those who received below-average scores were thought to have poor practice. On one of the questions, respondents could give multiple responses, so the maximum score that a pet owner could score was nine, whereas it was six for respondents not owing any pets.

### 2.4. Data Entry and Analysis

The data obtained from the interviews were analyzed using Statistical Package for Social Sciences (SPSS) version 23. The data was filtered and validated for missing values and inconsistencies. Simple descriptive statistics was performed using the Microsoft Excel 2016. The Chi-square test (X^2^) was done to test the association between practices of respondents towards rabies and their sociodemographic characteristics. In case the assumptions of the Chi-square test were violated, Fisher’s Exact Test (FET) for a 2 × 2 contingency table and likelihood ratio (LR) for tables bigger than 2 × 2 tabulation was used. Phi and Cramers’ V was used to interpret the strength of association, as suggested by Akoglu [17]. A *p* value of less than 0.05 was judged as being significant.

### 2.5. Ethics Statement

The guidelines of the 2013 World Medical Association (WMA) Declaration of Helsinki Ethical Principles for Medical Research Involving Human Subjects was followed and the study was approved by the Research and Capacity Building Committee of Association of Nepalese Agricultural Professionals of Americas (NAPA). Furthermore, the research team followed the research protocols to safeguard the privacy and confidentiality of respondents and their voluntary participation of the respondents. These protocols included strictly seeking oral consent from the respondents that was read to the participants prior to administration of the survey. Furthermore, respondents were also informed that the privacy and confidentiality of their response and protection of their individual identity was being maintained at all times of the research process. The research team was mindful of the social, cultural, religious, and economic background of respondents at the time of designing research tools.

## 3. Results

### 3.1. Sociodemographic Characteristics of Respondents

There was a total of 308 respondents. The majority of the respondents were male (75.3%, 232/308). The average age of the respondents was 44.9 ± 11.8 years with an average of 7 years of education. Over half of respondents were of Madeshi ethnic background (51.6%, 159/308), and the rest were Aadibasi/Janajati, Brahmin, Chhetri, and Musalman. The majority of respondents (87.0%, 268/308) follow Hinduism. Other demographic details are in Table 1.

### 3.2. Pet and Domestic Animal Ownership

A relatively low percentage of respondents (22.7%, 70/308) kept pet animals. Out of a total 70 households keeping pets, 59 households kept dogs whereas 11 kept cats. There were 23 households in Siraha, 22 households in Parsa, and 25 households in Parasi that owned a pet. Half of the respondents (51.9%, 160/308) had livestock. Table 2 shows the number of households keeping animals including pet animals.

### 3.3. Purpose of Owing Pets and Housing of Pets

Descriptive statistics show that 33.9% (20/59) of respondents kept dogs for guarding their houses and properties (Table 3). Similarly, 28.6% of respondents kept dogs/cats for companionship. When inquired about housing of pets, 28.6% (20/70) of the respondents told they housed their pet animals in cages. A total of 25.7% (18/70) respondents let their pet animals to freely roam around.

## 4. Practices towards Rabies

### 4.1. Rabies Vaccination and Record Keeping

Out of 70 respondents owning pet animals in their house, 82.9% (58/70) of them vaccinated their dogs and cats against rabies (Table 4). Out of them, 87.9% (51/58) kept a record of vaccination. The Chi-square test shows a strong association between the practice of vaccinating their pet animals with the age of pet owners (X^2^ = 4.478, *p* = 0.034) and their level of education (LR = 20.138, *p* < 0.001). It shows that whether the pet animal owners vaccinate their pets is related to the age of pet owners and their educational level. It is very likely that pet owners with a higher education and in higher age bracket vaccinate their pets.

Descriptive statistics show that 74.3% (52/70) of respondents restricted their pets from roaming in their community. A weak association was found between the age of pet owners and the practice of restricting pets to roam outside (X^2^ = 4.781, *p* = 0.029). Similarly, there was a weak association between the level of education of pet owner and the practice of restricting pets to roam outside (X^2^ = 8.881, *p* = 0.012).

### 4.2. Treatment Seeking Behavior of Respondents

Almost all respondents said they visit the hospital if they are bitten by rabid or rabies suspected animals (Table 5). Similarly, 82.8% (255/308) of respondents knew that they should visit the hospital if they are scratched by stray dogs or cats. Proportionately more respondents in Parasi (90.4%, 94/104) and Parsa (89.2%, 91/102) knew that they should visit a hospital than those from Siraha (68.6%, 70/102). Only 67.2% (207/308) of respondents said they would immediately wash wounds with soap and water after the bite of animals. The Chi-square test showed a very strong relationship between districts (X^2^ = 23.609, *p* < 0.001), pet ownership (X^2^ = 6.726, *p* = 0.010), gender (X^2^ = 3.971, *p* = 0.046), and education level (X^2^ = 20.240, *p* < 0.001) with the practice of washing the bitten area with soap and water. Phi and Cramers’ V signifies that gender and pet ownership had a moderate, whereas district and education level has a very strong, association/relationship. It shows that people with a higher education level are more likely to wash bitten areas with soap and water.

Traditional beliefs among the respondents were also seen as 18.2% (56/308) of respondents said that they would visit traditional healers if they were bitten by a rabid animal or rabies suspected animal. Similarly, the Chi-square test found an association between districts (X^2^ = 51.349, *p* < 0.001), ethnicity (X*^2^* = 11.594, *p* = 0.021), main occupation (X*^2^* = 12.166, *p* = 0.016), and level of education (X*^2^* = 20.403, *p* < 0.001) with the practice of going to a traditional healer after a bite. District and the level of education had a very strong relationship, whereas ethnicity and main occupation had a strong relationship, to seeking help of traditional healers.

### 4.3. Actions Taken on Rabid Animals/Rabies Suspected Animals

Approximately half of the respondents (50.6%, 156/308) reported that they usually kill rabies suspected or rabid animals if they bite people. Only 28.6% (88/308) of respondents shared that they would tie/cage such animals so that they will not cause harm to other people. Similarly, 20.8% (64/308) of respondents said that they will not carry out any of the above steps. The Chi-square test of association revealed that there was an association between districts (X^2^ = 10.234, *p* = 0.006), pet ownership (X^2^ = 26.180, *p* < 0.001), main occupation (X^2^ = 12.333, *p* = 0.015), level of education (X^2^ = 17.443, *p* < 0.001), and household income (X^2^ = 13.258, *p* < 0.001) with the practice of tying or caging a presumed rabid animal by a respondent.

### 4.4. Practice of Informing Authorities

Approximately 60% (184/308) of the respondents stated that they would not bother to inform/report to the concerned authorities if they see someone bitten by a presumed rabid dog or if they find the behavior of an animal to resemble rabies.

The Chi-square test of association revealed that there was an association between districts (X^2^ = 22.109, *p* < 0.001), pet ownership (X^2^ = 10.736, *p* = 0.001), ethnicity (X^2^ = 18.162, *p* = 0.001), main occupation (X^2^ = 25.413, *p* < 0.001), income level (X^2^ = 26.334, *p* < 0.001), education level (X^2^ = 40.865, *p* < 0.001), and family size (X^2^ = 10.175, *p* = 0.006) with dependent variables, i.e., ‘practice of informing concerned authorities if the respondent saw someone bitten by a presumed rabid dog’. Similarly, the Chi-square test of association revealed that there was an association between districts (X^2^ = 22.109, *p* < 0.001), pet ownership (X^2^ = 10.736, *p* = 0.001), ethnicity (X^2^ = 18.162, *p* = 0.001), main occupation (X^2^ = 25.413, *p* < 0.001), income level (X^2^ = 26.334, *p* < 0.001), education level (X^2^ = 40.865, *p* < 0.001), and family size (X^2^ = 10.175, *p* = 0.006) and the ‘practice of reporting concerned authorities if the respondent found the behavior of an animal to resemble rabies’. For both dependent variables, district, main occupation, income level, and level of education had a very strong relationship, whereas pet ownership, ethnicity, and family size had a strong relationship. It shows that most demographic characteristics of respondents have influence on their practices or behaviors towards rabies.

### 4.5. Association between the Practice Category of Respondents and Sociodemographic Traits

The practice category of respondents significantly varied according to their demographic characteristics (Table 6). The Chi-square test revealed that there was a very strong association between independent variables (districts (X^2^ = 25.562, *p* < 0.001), family size (X^2^ = 16.010, *p* < 0.001), household income (X^2^ = 20.753, *p* < 0.001), and education level (X^2^ = 41.485, *p* < 0.001)) and the dependent variable: practice level associated with rabies. Similarly, there was a strong association between independent variables (pet ownership (X^2^ = 7.052, *p* = 0.008), ethnicity (X^2^ = 11.409, *p* = 0.022), and a main occupation (X^2^ = 17.448, *p* = 0.002)) and practice level associated with rabies. There was a moderate association between respondents’ household head’s gender and practice level associated with rabies (X^2^ = 5.299, *p* = 0.022).

## 5. Discussion

Rabies is a serious public health concern, which is mostly reported in Asian and African countries. It is critical that people, for example, HHs, have the necessary skills and knowledge to prevent rabies and respond to animal bites. Understanding what community members are practicing and what their views are of treatment-seeking behavior are crucial for human rabies prevention. Our research has attempted to provide insights into these aspects, specifically practices that people follow towards rabies in Nepal.

Our study findings that most of the respondents keep pet animals (dogs/cats) in their houses for guarding and companionship is in contrast to a study in Mozambique where the main reason for keeping such pet animals was to protect crops from the attack of monkeys (68.8%) [18]. The finding that the majority of respondents confined their pets indoors or in a cage resonates with a study in India [14] where 84% of dogs were confined, but it is in contrast to that of Ethiopia where dogs were left free [19]. It is known that dogs that are allowed to roam free are more prone to contact rabid animals and acquire rabies infection [20,21].

Awareness of community members to vaccinate their pets is paramount for rabies prevention and control [22]. According to Centers for Disease Control and Prevention (CDC) [22], vaccines can be used for pre and post exposure care. People with a higher risk of rabies exposure, such as those working in veterinary hospitals or bush meat markets or those who are likely to be exposed to potentially infected animals, are advised to take the pre-exposure vaccine. People who are exposed to rabies or rabies suspected cases are recommended to take the post exposure anti-rabies vaccination.

Pre-exposure prophylaxis vaccination of dogs, cats, ferrets, and livestock is one of the recommended methods to stop virus spill over from wild animals to domestic animals and then to humans. Animal (dogs, cats, ferrets) owners should be aware of the fact that animals can be vaccinated as early as three months of age and given a booster after every year. In humans, PEP should be started soon after the bites or exposure to rabies suspected animals. The sooner such a vaccination occurs, the better the prognosis. Knowing the vaccination schedules, side effects, if any, of vaccines, along with the availability and accessibility and cost of vaccines, are crucial for rabies control and prevention.

A good proportion of respondents (82.9%, 58/70) had their pets vaccinated against rabies was found in our study, which is better than in studies conducted elsewhere in the world [19,23,24,25,26,27]. Among pet owners who were vaccinating their pets, some of them were not aware of the age at which a dog could receive vaccination for the first time. One fifth of pet owners did not vaccinate their pets. This could be due to a number of reasons, including not knowing the importance and schedule of vaccination, unavailability of vaccines, high vaccination fee, and vaccination points located far away from their places. In addition, the reason for the low percentage of people administrating ARV might be due to the lack of rabies control programs.

One in ten pet owners did not keep vaccination record of their pets. Therefore, whenever vaccination camps and vaccination is carried out, the vaccinators should emphasize to pet owners the significance of keeping the vaccination record as this helps in improving rabies control and eradication effectiveness.

The use of soap and water to clean rabies-infected wounds or bitten areas for at least 15 min can increase survival of affected animals by 50% [28]. Applying this lifesaving, cheap, readily and easily available first aid treatment is critical to prevent and control rabies from spreading. Two thirds of the respondents in our study doing first aid at their homes is better than that done in in Kathmandu, Nepal (10%) in 2021 [29], Rwanda (20.4%) [30], Cameroon (6%) [31], and Tanzania [26]. Gebremeskel et al. [32], in Ethiopia, found that the majority of respondents (92.4%) would cleanse their wounds with soap and water. This difference in practice could be due to the difference in the level of awareness in the communities. In our study, some respondents also bandaged and applied povidone iodine to clean and treat the bitten areas similar as reported by Ntampaka and colleagues [30].

Understanding how rabies is transmitted from animals to animals and from animals to humans and the factors contributing to such transmission are critically important to prevent and control rabies and loss of animal and human life. For example, according to the WHO [1], “education on dog behavior and bite prevention for both children and adults is an essential extension of rabies vaccination programs and can decrease both the incidence of human rabies and the financial burden of treating dog bites”.

Our study showing that almost all the respondents visit the hospital if they are bitten by a presumed rabid dog or rabies suspected animal, which resonates with previous studies in Kathmandu, Nepal, that 87.3% respondents would visit to hospitals [29], and by Sambo et al. [26], showing 83% respondents would seek medical attention if bitten by such rabies infected or suspected animals.

The practice of seeking services from a spiritual healer for a suspected rabies victim has also been documented. The results are a matter of concern since about one fifth of respondents in our study still believe in traditional healers and opt to seek their help when bitten by rabid or rabid suspected animals. However, reliance on traditional healers was also found in studies conducted elsewhere [19,33,34,35,36,37,38,39,40,41,42,43]. Traditional methods of treating rabid or rabies suspected cases are not scientifically proven and not recommended and people should be cautioned of this. Eighty-seven percent of respondents in India were aware that traditional practices are ineffective against rabies treatment [14]. Moreover, only 9.7% of respondents in Cameroon would consult traditional healers [31]. The preference of traditional healing methods may be due to a lack of knowledge or belief, and an easy access to traditional medicine and traditional healers. The gap in the knowledge can keep the communities at risk of rabies and can be responsible for majority of human death.

Half of the respondents in our study stated that the practice of killing rabid or rabies suspected animals if they bite people, which is similar in other countries and settings: 47.4% in Kathmandu [29], 79% in Tanzania [26], 71.1% in Ethiopia [40], 23.7% in North West Ethiopia [19], 54.3% in Morocco [41], and 63.2% in Pakistan [27].

Nearly three in ten respondents said they would cage rabid or rabies suspected animals if they bite people. In Indonesia, only a small number of respondents (13%) believed that presumed rabid dogs should be caught [24]. The low proportion of respondents indicating that they would prefer caging presumed rabies or rabid animals might be because they want to avoid contracting the rabies virus while handling them. Keeping suspected dogs in isolation for 10 days (quarantine) to verify if they had rabies or not is highly recommended irrespective of the dog vaccination status and is crucial for rabies prevention and control. This practice is gaining popularity in Africa as a study showed that 69% of respondents in Rwanda followed this method [30]. Inhuman ways of killing rabid or rabies suspected animals, including using axes, swords, and bamboo sticks, was also reported in our study; however, some respondents denied killing of dogs as it may cause sin.

Six in ten respondents said that they would not bother to inform a concerned authority if they see someone bitten by a presumed rabid dog or find the behavior of an animal to be similar to rabies. This is in contrast to Chaudhary and Dangi [29] in Kathmandu, Nepal, who found that almost all (98.3%) respondents would inform public health officials of any rabies outbreak, and is similar to Tiwari et al. [14], who found that 73% of respondents would report it to municipal authority. In a study by Sambo et al. [26], only 7% of respondents claimed that they would report any rabies-related incident to the concerned authority, which is far lower than our study. Unwillingness to report suspected cases and bites makes it difficult for concerned health authorities to comprehend the extent of the problem and take necessary preventative measures. People should be made aware of the fact that it is their duty to report such cases to the concerned authorities. Improved veterinary surveillance in affected areas as well as public education about rabies can help with better reporting and vaccination coverage in dogs [44].

The results of this study demonstrate that various demographic variables viz. district, pet ownership, ethnicity, family size, main occupation, level of education, and HH income were significantly associated with rabies practices. It is likely that pet owners are more knowledgeable on practice towards rabies than other people. They have continuous exposure to veterinary clinicians and rabies vaccination camps, and gain information on the timing of vaccination and how to care for dogs/cats and prevent exposure to rabies. Wolelaw et al. [16] found that the respondents aged between 18 to 29 were more likely to practice good rabies preventive and control methods than those over 45. We can assume that people with a higher level of education follow good practices towards rabies as they read and hear more in the media and from other community members about the present condition and how to control rabies than others do. Respondents with higher monthly income were more likely to have a good practice score than low income respondents found in our study, which resonates with research in Ethiopia [16] and the KAP Study conducted in Bahir Dar town [45]. This may be because members of the community who earn a high or middle household income frequently interact with professional people, including animal health workers. During this time, they may have learned about rabies.

Changes in livestock management practices, such as limited or no grazing in open public pasture lands, may be an option where rabid or rabies suspected animals come into contact with domestic animals, and the virus may spill over to healthy animals. Proper housing of livestock to avoid rabies exposures from stray dogs or carrier animals is also recommended. Freely letting animals to roam with little and no monitoring will also increase chances of being exposed to stray dogs and wild animals, which are reservoirs of rabies.

Our study has some limitations. First, the study was done employing snowballing sampling and there may be selection bias in the results. Second, the data were collected from HHs only. Understanding children’s knowledge levels is important since targeted education of this group can lead to long-term community changes.

## 6. Conclusions

While there are several important messages we could draw about the practices of the household heads towards rabies in rural Nepal from this study, some respondents indicated their unwillingness to report to the concerned authorities the rabies or rabies suspected cases in their communities, some said they still rely on traditional healers for the treatment of rabies, some owners indicated they do not vaccinate their pet animals and the pet owners did not keep vaccination records, which are of great concern. This indicates apparent gaps in information and knowledge among rural people about rabies. Interestingly, many practices towards rabies are found to be associated with the socio-demographic characteristics of respondents. Future research should focus on examining how practices of people towards rabies prevention and control are influenced by those demographic attributes, such as gender, pet ownership, ethnicity, household’s gender, family size, occupation, education, household income, and age of the respondents.

A consorted effort to raise awareness of rabies and good practices towards rabies in household heads in rural Nepal would be critical. Education and awareness campaigns about the importance of pre- and post-exposure prophylaxis against rabies are of paramount importance. Regular assessment of knowledge and behavior of owners towards vaccinating their pets against rabies should be part of a master plan for rabies eradication. Community members need education about animal behavior, particularly pet and rabid or rabies suspected animals, how to protect themselves from being bitten by such animals, and how to respond in the event of a bite.

## Figures and Tables

**Table 1:** Socio-demographic characteristics of respondents.

Socio-Demographics		Siraha, *n* = 102	Parsa, *n* = 102	Parasi, *n* = 104	Total, *n* = 308
Gender	Male	72 (70.6%)	89 (87.3%)	71 (68.3%)	232 (75.3%)
	Female	30 (29.4%)	13 (12.7%)	33 (31.7%)	76 (24.7%)
Age	Lowest to 40 (≤40)	46 (45.1%)	45 (44.1%)	41 (39.4%)	132 (42.9%)
	41 to highest (≥41)	56 (54.9%)	57 (55.9%)	63 (60.6%)	176 (57.1%)
Family Size	Mean ± SD	6.74 ± 2.3	6.22 ± 2.4	6.18 ± 2.7	6.4 ± 2.5
Household head gender	Male	96 (94.1%)	100 (98.0%)	80 (76.9%)	276 (89.6%)
	Female	6 (5.9%)	2 (2.0%)	24 (23.1%)	32 (10.4%)
Ethnicity	Madeshi	58 (56.9%)	64 (62.7%)	37 (35.6%)	159 (51.6%)
	Aadibasi/Janajati	20 (19.6%)	17 (16.7%)	17 (16.3%)	54 (17.5%)
	Brahmin	9 (8.8%)	10 (9.8%)	32 (30.8%)	51 (16.6%)
	Chhetri	6 (5.9%)	8 (7.8%)	10 (9.6%)	24 (7.8%)
	Musalman	9 (8.8%)	3 (2.9%)	8 (7.7%)	20 (6.5%)
Religion	Hinduism	89 (87.3%)	91 (89.2%)	88 (84.6%)	268 (87.0%)
	Islam	9 (8.8%)	3 (2.9%)	8 (7.7%)	20 (6.5%)
	Buddhism	4 (3.9%)	8 (7.8%)	7 (6.7%)	19 (6.2%)
	Christianity	0	0	1 (1.0%)	1 (0.3%)
Years of education	No formal education (0 years of education)	25 (24.5%)	15 (14.7%)	22 (21.2%)	62 (20.1%)
	School level (1 to 10)	45 (44.1%)	72 (70.6%)	49 (47.1%)	166 (53.9%)
	College/University (11 to highest)	32 (31.4%)	15 (14.7%)	33 (31.7%)	80 (26.0%)
Main family occupation	Self-employed/Own business	46 (45.1%)	31 (30.4%)	35 (33.7%)	112 (36.4%)
	Agriculture	36 (35.3%)	35 (34.3%)	27 (26.0%)	98 (31.8%)
	Private Jobs	8 (7.8%)	17 (16.7%)	12 (11.5%)	37 (12.0%)
	Government Job/Public Services	5 (4.9%)	6 (5.9%)	20 (19.2%)	31 (10.1%)
	Others	7 (6.9%)	13 (12.7%)	10 (9.6%)	30 (9.7%)
Monthly Household income of family	Lower (Rs. 10,000 to Rs. 25,000)	61 (59.8%)	74 (72.5%)	41 (39.4%)	176 (57.1%)
	Middle/Upper (Rs. 25,001 to Rs. 60,000)	41 (40.2%)	28 (27.5%)	63 (60.6%)	132 (42.9%)

Note: Rs. refers to Nepalese currency in rupees.

**Table 2:** Households with pet and domestic animal ownership in the study area.

Pet and/or Domestic Animals Owned	Siraha, *n* = 102	Parsa, *n* = 102	Parasi, *n* = 104	Total, *n* = 308
Dogs	21 (20.6%)	19 (18.6%)	19 (18.3%)	59 (19.2%)
Cats	2 (2.0%)	3 (2.9%)	6 (5.8%)	11 (3.6%)
Cattle	22 (21.6%)	12 (11.8%)	4 (3.8%)	38 (12.3%)
Buffalo	35 (34.3%)	38 (37.3%)	17 (16.3%)	90 (29.2%)
Goats	63 (61.8%)	56 (54.9%)	19 (18.3%)	138 (44.8%)
Poultry	13 (12.7%)	11 (10.8%)	11 (10.6%)	35 (11.4%)
Pigs	3 (2.9%)	4 (3.9%)	1 (1.0%)	8 (2.6%)

**Table 3:** Purpose of keeping pet animals and their housing types.

The Main Purpose of Owning a Dog/Cat	Siraha, *n* = 23 pets	Parsa, *n* = 22 pets	Parasi, *n* = 25 pets	Total, *n* = 70
Guarding (*n* = 59, dogs)	8/21 (38.1%)	3/19 (15.8%)	9/19 (47.4%)	20/59 (33.9%)
Companionship	8/23 (34.8%)	9/22 (40.9%)	3/25 (12%)	20/70 (28.6%)
Family/children wish	3/23 (13.0%)	6/22 (27.3%)	7/25 (28%)	16/70 (22.9%)
Hobby	4/23 (17.4%)	1/22 (4.5%)	5/25 (20%)	10/70 (14.3%)
Other reasons	0	3/22 (13.6%)	1/25 (4.0%)	4/70 (5.7%)
**Housing for dog/cat**				
Housed in cages	6/23 (26.1%)	4/22 (18.2%)	10/25 (40.0%)	20/70 (28.6%)
Tied outside the house (*n* = 59, dogs)	9/21 (42.9%)	9/19 (47.4%)	2/19 (10.5%)	20/59 (33.9%)
Free living inside the house	2/23 (8.7%)	5/22 (22.7%)	5/25 (20.0%)	12/70 (17.1%)
Free to roam around	6/23 (26.1%)	4/22 (18.2%)	8/25 (32.0%)	18/70 (25.7%)

**Table 4:** Vaccination of pets and record keeping of vaccination by pet owners.

Vaccination and Record Keeping	Siraha, *n* = 23 pets	Parsa, *n* = 22 pets	Parasi, *n* = 25 pets	Total, *n* = 70
Do you vaccinate your dog/cat against rabies? (*n* = 70, respondents having pet animal)				
Yes	20/23 (87.0%)	19/22 (86.4%)	19/25 (76.0%)	58/70 (82.9%)
Do you keep record of rabies vaccination of your pet animals? (*n* = 58, respondents vaccinating their pets)				
Yes	18/20 (90.0%)	18/19 (94.7%)	15/19 (78.9%)	51/58 (87.9%)
Do you restrict your pet(s) to roam in the community? (*n* = 70, respondents having pet animal)				
Yes	17/23 (73.9%)	18/22 (81.8%)	17/25 (68.0%)	52/70 (74.3%)

**Table 5:** Response to different practices related questions.

Practices Related Questions	Siraha, *n* = 102	Parsa, *n* = 102	Parasi, *n* = 104	Total, *n* = 308
What immediate action(s) do you take after being bitten by rabid or rabies suspected animal?				
Washing with soap and water	77 (75.5%)	79 (77.5%)	51 (49%)	207 (67.2%)
Go to traditional healer	41 (40.2%)	11 (10.8%)	4 (3.8%)	56 (18.2%)
Visit hospital	102 (100.0%)	102 (100.0%)	103 (99.0%)	307 (99.7%)
Will you visit hospital if you are scratched by stray dog/cat?				
Yes	70 (68.6%)	91 (89.2%)	94 (90.4%)	255 (82.8%)
No	32 (31.4%)	11 (10.8%)	10 (9.6%)	53 (17.2%)
What action do you take on presumed rabid animal after it bites people?				
Tie/cage	20 (19.6%)	27 (26.5%)	41 (39.4%)	88 (28.6%)
Kill	38 (37.3%)	64 (62.7%)	54 (51.9%)	156 (50.6%)
Do nothing	44 (43.1%)	11 (10.8%)	9 (8.7%)	64 (20.8%)
Do you inform concerned authorities if you see someone bitten by a presumed rabid dog?				
Yes	31 (30.4%)	32 (31.4%)	61 (58.7%)	124 (40.3%)
No	71 (69.6%)	70 (68.6%)	43 (41.3%)	184 (59.7%)
Will you report to concerned authority if you find the behavior of dog/animal resembling to rabies?				
Yes	31 (30.4%)	32 (31.4%)	61 (58.7%)	124 (40.3%)
No	71 (69.6%)	70 (68.6%)	43 (41.3%)	184 (59.7%)

**Table 6:** Association of different socio-demographic characteristics with practice category of respondents.

Socio-Demographics		Practice Category		X*^2^*	df	*p* Value
		Poor Practice	Good Practice			
Districts	Siraha	74	28	22.562	2	<0.001 ***
	Parsa	66	36			
	Parasi	43	61			
Gender	Male	141	91	0.721	1	0.396
	Female	42	34			
Pet ownership	Yes	32	38	7.052	1	0.008 **
	No	151	87			
Ethnicity	Brahmin	23	28	11.409	4	0.022 *
	Chhetri	15	9			
	Adhibashi/Janajati	27	27			
	Madhesi	102	57			
	Mushalman	16	4			
Household head	Male	170	106	5.299	1	0.022 *
	Female	13	19			
Family size	Small (1 to 5)	58	68	16.010	2	<0.001 ***
	Medium (6 to 10)	109	51			
	Large (≥11)	16	6			
Religion	Hinduism	154	114	-	-	-
	Buddhism	13	6			
	Islam	16	4			
	Christianity	0	1			
Main occupation	Agriculture	65	33	17.448	4	0.002 **
	Government/Public Service	8	23			
	Self/Own employed	69	43			
	Private Jobs	21	16			
	Others	20	10			
Level of education	No formal education (0 years of education)	51	11	41.485	2	<0.001 ***
	School level (1 to 10)	107	59			
	College/University (11 to highest)	25	55			
Household income	Lower (10,000 to 25,000)	124	52	20.753	1	<0.001 ***
	Middle/Upper (25,001 to 60,000)	59	73			
Age group	Lowest to 40 (≤40)	73	59	1.620	1	0.203
	41 to highest (≥41)	110	66			
History of Animal bite (Dog/cat/fox) to you and your family	Yes	50	36	0.081	1	0.777
	No	133	89			

Note: * *p* < 0.05, ** *p* < 0.01, *** *p* < 0.001, df = degrees of freedom.

## Data Availability

The data used to support the findings of this study are available from the corresponding author upon reasonable request.

## Supplementary Materials

The following supporting information can be downloaded at: https://www.mdpi.com/article/10.3390/ijerph20075427/s1, Supplementary File S1: Households’ practices towards rabies prevention and control in rural Nepal.

## References

[1] WHO Rabies. https://www.who.int/news-room/fact-sheets/detail/rabies.

[2] Singh R., Singh K.P., Cherian S., Saminathan M., Kapoor S., Reddy G.B.M., Panda S., Dhama K. (2017). Rabies—Epidemiology, Pathogenesis, Public Health Concerns and Advances in Diagnosis and Control: A Comprehensive Review. Vet. Q..

[3] WHO (2018). Zero by 30: The Global Strategic Plan to End Human Deaths from Dog-Mediated Rabies by 2030.

[4] FAO Towards a Rabies-Free World as Unparalleled Global Initiative Gets Underway. https://www.fao.org/news/story/en/item/1040394/icode/.

[5] Wilde H., Lumlertdacha B., Meslin F.X., Ghai S., Hemachudha T. (2016). Worldwide Rabies Deaths Prevention-A Focus on the Current Inadequacies in Postexposure Prophylaxis of Animal Bite Victims. Vaccine.

[6] FAO (2017). The Food and Agriculture Organization and Rabies Prevention and Control.

[7] (2020). Annual Report Department of Health Services 2019/2020.

[8] Acharya K.P., Adhikari N., Tariq M. (2020). Fight against Rabies in Nepal: Immediate Need for Government Intervention. One Health.

[9] EDCD (2019). National Guideline Rabies Prophylaxis in Nepal (2019).

[10] RVPL Introduction. http://www.rvpl.gov.np/.

[11] Pantha S., Subedi D., Poudel U., Subedi S., Kaphle K., Dhakal S. (2020). Review of Rabies in Nepal. One Health.

[12] CDC Rabies around the World. https://www.cdc.gov/rabies/location/world/index.html.

[13] Mission Rabies Mission Rabies. http://www.missionrabies.com/.

[14] Tiwari H.K., Vanak A.T., O’Dea M., Robertson I.D. (2019). Knowledge, Attitudes and Practices (KAP) towards Rabies and Free-Roaming Dogs (FRD) in Shirsuphal Village in Western India: A Community Based Cross-Sectional Study. PLoS Negl. Trop. Dis..

[15] Tenzin, Dhand N.K., Rai B.D., Changlo, Tenzin S., Tsheten K., Ugyen P., Singye K., Ward M.P. (2012). Community-Based Study on Knowledge, Attitudes and Perception of Rabies in Gelephu, South-Central Bhutan. Int. Health.

[16] Wolelaw G.A., Yalew W.A., Azene A.G., Wassie G.T. (2022). Rabies Prevention Practices and Associated Factors among Household Heads in Bure Zuria District, North West Ethiopia. Sci. Rep..

[17] Akoglu H. (2018). User’s Guide to Correlation Coefficients. Turkish J. Emerg. Med..

[18] Mapatse M., Sabeta C., Fafetine J., Abernethy D. (2022). Knowledge, Attitudes, Practices (KAP) and Control of Rabies among Community Households and Health Practitioners at the Human-Wildlife Interface in Limpopo National Park, Massingir District, Mozambique. PLoS Negl. Trop. Dis..

[19] Bihon A., Meresa D., Tesfaw A. (2020). Rabies: Knowledge, Attitude and Practices in and Around South Gondar, North West Ethiopia. Diseases.

[20] Tenzin T., Ahmed R., Debnath N.C., Ahmed G., Yamage M. (2015). Free-Roaming Dog Population Estimation and Status of the Dog Population Management and Rabies Control Program in Dhaka City, Bangladesh. PLOS Negl. Trop. Dis..

[21] Rinzin K., Tenzin T., Robertson I. (2016). Size and Demography Pattern of the Domestic Dog Population in Bhutan: Implications for Dog Population Management and Disease Control. Prev. Vet. Med..

[22] CDC Rabies VIS. https://www.cdc.gov/vaccines/hcp/vis/vis-statements/rabies.html.

[23] Hagos W.G., Muchie K.F., Gebru G.G., Mezgebe G.G., Reda K.A., Dachew B.A. (2020). Assessment of Knowledge, Attitude and Practice towards Rabies and Associated Factors among Household Heads in Mekelle City, Ethiopia. BMC Public Health.

[24] Widyastuti M.D.W., Bardosh K.L.ᅟ, Sunandar, Basri C., Basuno E., Jatikusumah A., Arief R.A., Putra A.A.G., Rukmantara A., Estoepangestie A.T.S. (2015). On Dogs, People, and a Rabies Epidemic: Results from a Sociocultural Study in Bali, Indonesia. Infect. Dis. Poverty.

[25] Kabeta T., Deresa B., Tigre W., Ward M.P., Mor S.M. (2015). Knowledge, Attitudes and Practices of Animal Bite Victims Attending an Anti-Rabies Health Center in Jimma Town, Ethiopia. PLoS Negl. Trop. Dis..

[26] Sambo M., Lembo T., Cleaveland S., Ferguson H.M., Sikana L., Simon C., Urassa H., Hampson K. (2014). Knowledge, Attitudes and Practices (KAP) about Rabies Prevention and Control: A Community Survey in Tanzania. PLoS Negl. Trop. Dis..

[27] Ahmed T., Hussain S., Zia U.U.R., Rinchen S., Yasir A., Ahmed S., Khan W.A., Tahir M.F., Ricketson R. (2020). Knowledge, Attitude and Practice (KAP) Survey of Canine Rabies in Khyber Pakhtunkhwa and Punjab Province of Pakistan. BMC Public Health.

[28] Radostits O.M., Gay C., Hinchcliff K.W., Constable P.D. (2006). Veterinary Medicine: A Textbook of the Diseases of Cattle, Horses, Sheep, Pigs and Goats.

[29] Chaudhary S., Dangi S. (2021). Awareness towards Rabies in the Residents of Kathmandu Metropolitan City, Nepal. J. Zoonotic Dis..

[30] Ntampaka P., Nyaga P.N., Gathumbi J.K., Tukei M., Niragire F. (2019). Knowledge, Attitudes and Practices Regarding Rabies and Its Control among Dog Owners in Kigali City, Rwanda. PLoS ONE.

[31] Costa G.B., Gilbert A., Monroe B., Blanton J., Ngam S.N., Recuenco S., Wallace R. (2018). The Influence of Poverty and Rabies Knowledge on Healthcare Seeking Behaviors and Dog Ownership, Cameroon. PLoS ONE.

[32] Gebremeskel A.K., Tanga B.M., Getachew A., Eshetu Y. (2019). Assessment of Public Knowledge, Attitude and Practices towards Rabies in the Community of Kombolcha, Southern Wollo, Amhara Reginal State, Ethiopia. J. Public Health Epidemiol..

[33] Jemberu W.T., Molla W., Almaw G., Alemu S. (2013). Incidence of Rabies in Humans and Domestic Animals and People’s Awareness in North Gondar Zone, Ethiopia. PLoS Negl. Trop. Dis..

[34] Mshelbwala P.P., Ogunkoya A.B., Maikai B.V. (2013). Detection of Rabies Antigen in the Saliva and Brains of Apparently Healthy Dogs Slaughtered for Human Consumption and Its Public Health Implications in Abia State, Nigeria. ISRN Vet. Sci..

[35] Digafe R.T., Kifelew L.G., Mechesso A.F. (2015). Knowledge, Attitudes and Practices towards Rabies: Questionnaire Survey in Rural Household Heads of Gondar Zuria District, Ethiopia. BMC Res. Notes.

[36] Ali A., Yimer E., Sifer D. (2013). A Study on Knowledge, Attitude and Practice of Rabies among Residents Abstract This Study Was Conducted in Addis Ababa during the Months of January and February, 2011 to Assess the Knowledge, Attitudes and Practices of the Communities on Rabies. A Cro. Ethiop. Vet. J..

[37] Gebeyaw S., Teshome D. (2016). Assessment of Knowledge, Attitude and Practice on Rabies in and Around Dessie City. Austin J. Vet. Sci. Anim. Husbandary.

[38] Tamirat K., Alemayehu L., Mulualem T. (2016). Community Perception towards Traditional Healers and Health Centers on Management of Dog Bites and Its Relation with Veterinary Public Health Activities. J. Vet. Sci. Anim. Husb..

[39] Pal P., Yawongsa A., Bhusal T.N., Bashyal R., Rukkwamsuk T. (2021). Knowledge, Attitude, and Practice about Rabies Prevention and Control: A Community Survey in Nepal. Vet. World.

[40] Nejash A., Boru M., Jemal J., Wezir A. (2017). Knowledge, Attitudes and Practices towards Rabies in Dedo District of Jimma Zone, Southwestern Ethiopia: A Community Based Cross-Sectional Study. Int. J. Med. Med. Sci..

[41] Bouaddi K., Bitar A., Bouslikhane M., Ferssiwi A., Fitani A., Mshelbwala P.P. (2020). Knowledge, Attitudes, and Practices Regarding Rabies in El Jadida Region, Morocco. Vet. Sci..

[42] Ghosh S., Chowdhury S., Haider N., Bhowmik R.K., Rana M.S., Prue Marma A.S., Hossain M.B., Debnath N.C., Ahmed B.N. (2016). Awareness of Rabies and Response to Dog Bites in a Bangladesh Community. Vet. Med. Sci..

[43] Krishnamoorthy Y., Vijayageetha M., Sarkar S. (2018). Awareness about Rabies among General Population and Treatment Seeking Behaviour Following Dog-Bite in Rural Puducherry: A Community Based Cross-Sectional Study. Int. J. Community Med. Public Health.

[44] Rinchen S., Tenzin T.T., Hall D., Van Der Meer F., Sharma B., Dukpa K., Cork S. (2019). A Community-Based Knowledge, Attitude, and Practice Survey on Rabies among Cattle Owners in Selected Areas of Bhutan. PLoS Negl. Trop. Dis..

[45] Guadu T., Shite A., Chanie M., Bogale B., Fentahun T. (2014). Assessment of Knowledge, Attitude and Practices about Rabies and Associated Factors: In the Case of Bahir Dar Town. Glob. Vet..

