# Supplementary material for: Households’ Practices towards Rabies Prevention and Control in Rural Nepal
Source: Int J Environ Res Public Health. 2023 Apr 6;20(7):5427. doi: 10.3390/ijerph20075427 (PMC10094547; doi:10.3390/ijerph20075427)
Supplement: Supplementary file 1 [file ijerph-20-05427-s001.zip › ijerph-2207777-supplementary.pdf]

Households' practices towards rabies prevention and control in rural Nepal

ID: **Date:** **Address:** **Interviewer:**
GPS reading:

1. General questions

In this section you are asked about pet and domestic animals in your house. Pet animals refers to dogs and cats.

**1a**	Do you have any pet animal(s) in your house?<br>a. Yes ☐ (Go to 1b to specify the pets number)<br>b. No☐ (Go to 1d)			**1c**	Are there any domestic animal(s) at your household?<br>a. Yes ☐ (Go to 1d and specify their corresponding number)<br>b. No ☐ (Go to 2a)				
**1b**	1b1: Dog (n):<br>1b1a: Years of keeping (YOK):	1b2	Cat (n):<br>1b2a: YOK:	**1d**	1d1: Cattle:	1d2	Buffalo:	1d3	Goat:
					1d4: Poultry	1d5	Others:		

2. Practices towards Rabies

2a	Do you vaccinate your dog/cat against rabies? *(If you do not have pet animals, Skip to 2d)* a. Yes ☐ b. No ☐
2b	Do you keep record of Rabies vaccination of your pet animals? a. Yes ☐ b. No ☐
2c	Do you restrict your dog(s)/ cat(s) to roam in the community? a. Yes ☐ b. No ☐
2d	What immediate action(s) do you take after being bitten by a rabid or rabid suspected animal? *Select all that apply*.<br>a. Washing with soap and water ☐ b. Go to traditional healer ☐ c. Visit hospital ☐ d. Do nothing ☐ e. Other specify:____
2e	What action do you take on presumed rabid animal after it bites others? a. Tie/cage ☐ b. Kill ☐ c. Do noting d. Other specify: ________
2f	Do you inform concerned authorities if you see someone bitten by a presumed rabid dog/ animal?<br>a. Yes ☐ b. No ☐
2g	Will you report to concerned authority if you find the behavior of dog/ animal resembling to rabies?<br>a. Yes ☐ b. No ☐
2h	Will you visit the hospital if you are scratched by a stray dog/cat? a. Yes ☐ b. No ☐

3. Purpose of keeping pet and its housing

3a	What's the main purpose of owning a dog by you? *(**Skip** to 4 if you do not have pet animals) Select one.*<br>a. Guarding ☐ b. Companionship☐ c. Family/Children wish☐<br>d. Hobby ☐ e. Other: ________
3b	How do you house your dog? *Select all that apply*. a. Housed in cages☐ b. Tied outside the house☐
	c. Free living inside the house☐ d. Free to roam around ☐

4. History of street or presumed rabid dog/cat bite? (1=Yes, 2=No), Here ORSB= Other rabies susceptible animal bite

**Code**	Description	Presumed rabid dog/cat	**Code**	Street dog/cat	Code	ORSB	**Code**	Description	Presumed rabid dog/ cat	**Code**	Street dog/ cat	Code	ORSB
**44a**	You		**44c**		**44e**		**44g**	Pet animal		**44i**		**44k**	
**44b**	Your family members		**44d**		**44f**		**44h**	Domestic animal		**44j**		**44l**	

5. Demographics

5a	Your gender: Male ☐ Female ☐	5e	Your age (year eg. 45 year):
5b	Your marital status: Married ☐ Single ☐ Divorcee ☐ Other:	5f	Family size: 6g1. Male: 6g2.Female
5c	Gender of household head (if HHH is different than the interviewee):<br>a. Male ☐ b. Female ☐	5g	Number of children (<12 years) in your household:
5d	Your ethnicity: a. Brahmin ☐ b. Chhetri ☐ c. Aadibasi and Janajati (Newar, Tharu, Gurung, Rai, etc) ☐<br>d. Madeshi ☐ e. Musalman ☐ f.Others, Specify:		
5h	Which religion do you follow? a. Hinduism ☐ b. Buddhism ☐ c. Islam ☐ d. Christianity ☐ e. Others		
5i	Fill in the blanks. I have received ………………..years of education. OR informal eduction ☐		
5j	Main occupation of your family:<br>a. Agriculture b. Government job/public services c. Self-employed/Own business d. Private Jobs e. Other, specify:______		
5k	What is the monthly household income of your family?		

Q6. If you would like to share additional thoughts or issues about rabies you can do so here.

Thank you for your time!
